# Sex differences in MAGEL2 gene promoter methylation in high functioning autism - trends from a pilot study using nanopore Cas9 targeted long read sequencing

**DOI:** 10.1186/s12920-024-02053-9

**Published:** 2024-11-29

**Authors:** Jelte Wieting, Kirsten Jahn, Stefan Bleich, Maximilian Deest, Helge Frieling

**Affiliations:** 1 Department of Psychiatry, Social Psychiatry and Psychotherapy, Hannover Medical School, Carl-Neuberg-Str. 1, Hannover, 30625 Germany; 2 Laboratory for Molecular Neuroscience, Feodor-Lynen-Str. 35, Hannover, 30625 Germany; 3 Present Affiliation: Oberberg Fachklinik Weserbergland, Brede 29, Extertal-Laßbruch, 32699 Germany

**Keywords:** MAGEL2, High-functioning autism, Autism spectrum disorder, Adult, Sex differences, Long read sequencing, Nanopore Cas9 targeted sequencing, NCATS, Methylation, 5mC modification

## Abstract

**Background:**

MAGEL2 is an autism susceptibility gene whose deficiency has been associated with autism-related behaviors in animal models and in syndromic human autism spectrum disorders (ASDs) such as Schaaf-Yang syndrome, but has not been studied in the broader autism spectrum. Given the capabilities of long-read sequencing technologies, this pilot study used a targeted nanopore sequencing approach to simultaneously examine MAGEL2 DNA sequence and methylation in adults with high-functioning autism (HFA) compared to neurotypical controls (NC).

**Methods:**

Using DNA extracted from peripheral blood, Cas9-targeted nanopore DNA sequencing was used to analyze MAGEL2, including its entire regulatory construct (chr15:23639316–23651466), for sequence variation and 5-methyl-cytosine (5mC) modification in a cohort of adults with HFA compared to sex- and age-matched NC. Given the known sex differences in ASD and MAGEL2 KO animal models, results were further analyzed by sex.

**Results:**

20 adults with HFA (10 males, 10 females) and 20 NC were included. While there were no overall differences in MAGEL2 DNA sequence and 5mC modification between HFA and NC, we found a significant difference in MAGEL2 gene promoter methylation between males and females with HFA and NC of both sexes, with HFA males tending to show hypomethylation in a 300 bp long differentially methylated region (chr15:23647640–23647939) around the MAGEL2 transcription start site.

**Conclusions:**

In this pilot study utilizing nanopore Cas9 targeted DNA sequencing, significant sex-specific differences in MAGEL2 gene promoter methylation were identified in male adults with HFA in comparison to control groups, suggesting the potential for sex-specific epigenetic differences. However, further replication in larger cohorts is required to validate these findings.

**Supplementary Information:**

The online version contains supplementary material available at 10.1186/s12920-024-02053-9.

## Background

Autism Spectrum Disorders (ASD) are characterized by persistent deficits in social interaction and communication, and by restricted, repetitive patterns of behavior, interests, and activities. While a proportion of 10–20% of ASDs are due to identifiable monogenic or oligogenic causes, complex polygenic inheritance is assumed the cause in idiopathic cases. Known for its association with Prader-Willi syndrome (PWS), genetic abnormalities in the human 15q11-13 chromosomal region, which is subject to imprinting effects, are a common cause of syndromic autism, accounting for approximately 1% of ASD cases [1]. This 15q11-13 chromosomal region contains several genes, including MAGE family member L2 (MAGEL2). In contrast to PWS, Schaaf-Yang syndrome (SYS) is caused solely by truncating mutations of this MAGEL2 gene. Different variants (deletions, duplications, transitions) cause a rather heterogeneous phenotype depending on the different effects due to the position and type of MAGEL2 variants [2]. However, in addition to some phenotypic overlap with PWS, there is a distinct autistic phenotype in SYS [3]. In addition, autism-like behaviors can be observed in MAGEL2 knockout (KO) mouse models. For example, MAGEL2 KO mice showed increased anxiety in novel environments and altered social interaction phenotypes [4] as well as deficits in social memory [5]. In general, alterations in MAGEL2 appear to be associated with behavioral changes across species, which may reflect abnormal production and processing of neuropeptides such as oxytocin [6]. As there are phenotypic sex differences in human ASD, especially in the high-functioning spectrum [7], previous studies also found behavioral changes in MAGEL2 KO rodent models, that were sometimes observed only in male animals [5]. However, a conclusive neurobiological explanation for the association of MAGEL2 alterations with autistic symptoms has not yet been found. Furthermore, to our knowledge, MAGEL2 alterations and their association with autistic symptoms have not been investigated in human autism case-control samples.

Third-generation long read DNA sequencing methods, such as nanopore sequencing, allow us to capture the DNA sequences of large gene segments or entire genes. Moreover, one can simultaneously examine them for sequence variants and epigenetic modifications without the need for prior amplification or modification of the DNA material for epigenetic studies, such as bisulfitation, which can potentially cause significant damage to the DNA material [8].

Given that de novo loss-of-function single nucleotide variants (SNVs) or insertion/deletion (indel) variants are significantly more common in individuals with ASD than in neurotypical matched controls [9], we wondered whether newly identified sequence variants of the MAGEL2 gene might also be detectable in the broader autism phenotype represented by a cohort of adults with high functioning autism (HFA).

In addition to sequence variation, epigenetic modifications strongly influence gene regulation. One of the most important epigenetic regulatory mechanisms is 5-methyl-cytosine (5mC) modification, in which a methyl group is added to the 5-carbon position of the cytosine ring in the context of CpG dinucleotides (cytosine followed by guanine, linked by a phosphate) within the DNA sequence. Most enriched within the gene promoter region (usually around the transcription start site (TSS)), the 5mC modification is commonly inversely associated with transcriptional activity and gene expression. DNA methylation patterns in autism susceptibility genes have been shown to be shared between idiopathic and syndromic forms of ASD [10].

Given the capabilities of long-read sequencing technologies, the purpose of this pilot study was to simultaneously examine MAGEL2 DNA sequence and methylation in adults with high-functioning autism (HFA) compared to neurotypical controls (NC) using a nanopore Cas9 targeted sequencing (nCATS) approach to elucidate the relationship between MAGEL2 (epi)genetic variation and HFA conditions.

## Methods

### Recruitment

The study included 20 individuals diagnosed with ICD-10 code F84.5 (Asperger syndrome) according to ICD-10 criteria at the time of recruitment. The diagnostic procedure followed the german diagnostic guideline for ASD [11], which was applied within the framework of ICD-10. All participants were diagnosed with Asperger syndrome using the Adult Asperger Assessment (AAA) [12], which is more rigorous than ICD-10. We chose the term “high-functioning autism” (HFA) for clarity, recognizing that Asperger’s has been replaced by the broader term ASD in ICD-11. However, HFA, although unofficial, conveys the specific ASD subset analyzed here.

Participants were selected from a larger registry study, Neurobiology in the Development of Mental Disorders. Diagnostic instruments included the Autism Quotient (AQ), the Empathy Quotient (EQ), and the Wechsler Adult Intelligence Scale - IV (WAIS-IV) [13]. Eligibility was confirmed by at least two experienced clinicians. Subjects met diagnostic criteria (ICD-10 *and* AAA), had an IQ > 70, and were aged 18–65. Non-autistic, healthy controls (NC) were matched for sex, age (± 5 years), and IQ (± 15 points) and underwent the same assessments except for the AAA interview. The final 40 participants were selected to ensure sex balance and to match age and intelligence to NC. All participants were recruited from an outpatient center specializing in ASD diagnosis.

The study protocol adhered to the Declaration of Helsinki and was prospectively reviewed and approved by the ethics committee of Hannover Medical School, Germany, under approval number 3054 − 2016. All subjects gave written informed consent to participate in the study. The mental health professionals in charge of the study have determined that all participants are capable of providing informed consent for their involvement in the research presented in this manuscript.

As this is a pilot study and there are no adequate previous studies for comparison, no prior sample size estimation was carried out (comments on the implications of this are provided in the Limitations section).

### Sampling

EDTA (ethylenediaminetetraacetic acid) blood samples were collected from both HFA and NC subjects by peripheral venipuncture. DNA extraction from EDTA blood was performed by the Hannover Unified Biobank using the ChemagicStar DNA-Blood1k kit (PerkinElmer Chemagen Technology, Baesweiler, Germany) on a Hamilton ChemagicStar (Hamilton Germany Robotics, Gräfelfing, Germany).

### DNA sequencing

Nanopore Cas9-targeted sequencing (nCATS) was used for DNA sequencing [14]. Thereby, CRISPR RNA (crRNA) protospacers are designed for specific Cas9 binding to cleave DNA at sites similar to the crRNA sequences. To that end, Cas9 further requires the Protospacer Adjacent Motif (PAM), adjacent to the 3’ end of the target sequence.

MAGEL2 sequence-specific CRISPR RNAs (crRNA) were designed using Alt-R Custom Cas9 crRNA Design Tool (IDT^®^, Coralville, IA, USA) and produced by IDT^®^, spanning the whole MAGEL2 sequence and including all associated gene regulatory units (chr15: 23639316–23651466). For optimization of coverage a total of four crRNAs were positioned over the MAGEL2 region, resulting in two fragments, but also a 360 bp blind spot from chr15:23647088–23,647,448.

Cas9 loaded with sequence-specific crRNA forms a ribonucleoprotein complex with catalytic trans-activating CRISPR RNA (tracrRNA), searching the genomic DNA sample for the target region using the crRNA “guide” sequence. If the crRNA matches and base-pairs with the target sequence, Cas9 cleaves both strands of the target sequence 3 bp upstream of the PAM. General principles of crRNA choice, which we followed also in the current study, are described in more detail in Gombert & Jahn et al. [15].

A table with the sequences, genomic position and PAMs of the crRNAs used for MAGEL2 nCATS is provided in Supplementary Material S1.

For DNA library preparation, Cas-mediated PCR-free enrichment was performed according to the protocol provided by the manufacturer Oxford Nanopore Technologies (Oxford, UK) with minor adjustments. The complete log is available for review on request from the manufacturer. Products were all purchased from Oxford Nanopore Technologies, Oxford, UK. Our adjustments to the protocol based on our experience during establishment phase are explained below.

Cas9 cleavage reaction was incubated at 37°C for 30 min and 72°C for 7 min as a slight deviation from the manufacturer’s protocol. Moreover, deviating from the original protocol, we used an amount of 0.6 µl Cas9 instead of 0.4 µl to optimize subsequent sequencing coverage. A unique barcode was selected for each sample and 5 samples were used within one flow cell. We used 5 µl instead of 3 µl barcode per sample as it showed an increased coverage during test runs.

Nanopore sequencing was performed on R9.4.1 flow cells using a MinION nanopore sequencer and Nanopore MinKnow Sequencing software. These flow cells contain a series of protein nanopores embedded in an electroresistant membrane. The electric current flowing through is measured at each individual nanopore. When a DNA molecule passes through a nanopore, the current is interrupted, and a characteristic pattern is created in dependence of the individual base occupying the pore in that moment. This pattern is then decoded using base and methylation calling algorithms to determine the DNA sequence and modifications.

### Data analyis

Nanopore raw reads (fast5 format) were basecalled using Guppy Basecalling Software Version 6.1.2 and aligned to the reference genome Hg38 using minimap2 [16].

Single nucleotide variants (SNVs) were called by longshot [17] using GenBank GCA_000001405.15 (GRCh38) as the reference sequence and a minimum allele frequency of 15 as the cut-off to determine genotypes (min_alt_count = 15). The output individual .vcf files per sample were merged using bcftools [18] and visualized in group comparison using the UCSC human genome browser [19]. The output was visually inspected for group-dependent clustering of any SNPs. We further looked explicitly for group differences in SNPs known to be associated with autism compared to ClinVar database [20] as well as novel SNPs within coding regions as well as gene regulatory units derived from ORegAnno [21].

Nanopolish was used for 5mC methylation calling. The getCoverage function of the bssseq package for R [22] was used to assess mean coverages by sample and individual CpG positions within the fragments. Coverage was visualized for distribution across samples and across genomic positions (Supplementary Material S2).

5-methyl cytosine modification probability was obtained and visualized in group comparison using NanoMethViz for R (plot_region function) [23]. BSSSeq type objects obtained via the NanoMethViz to BSSSeq interface were processed for statistical testing of differentially methylated loci (DML) and regions (DMR) using DSS for R [24]. To determine differential methylation, Wald tests for beta-binomial distribution were performed at each CpG site to determine DML and subsequent DMR. As the test statistic takes both biological variation (characterized by the dispersion parameter) and sequencing depth into account, the method is described as particularly suitable for small sample sizes (please also refer to our explanations in the Limitations section). Based on the test results, DMLs were called using the DSS callDML function. We examined our data for differences of > 5% betwen the groups in estimated mean methylation (flags p.threshold = 0.05, delta = 0.05), calculating the probability that the group difference in mean methylation is greater than delta 5% at a % significance level. DMR detection using the callDMR function is based on these DML test results. We applied the DSS default requirements for DMRs (minimum length of 50 bps, minimum number of CpGs of 3, minimum percentage of significantly group-different CpGs within the DMR of 50%). Here, we again used a group difference in mean methylation of at least 5% as theshold (flags p.threshold = 0.05, delta = 0.05). We performed additional analyses of sex differences in addition to HFA versus NC group comparisons, applying the same procedure.

The data sets generated and analyzed in this study are available from the European Genome Phenome Archive (EGA) at https://ega-archive.org/studies/EGAS50000000508. The analysis code used here is publicly available at https://github.com/wietingj/nCATS_analysis.

## Results

### Group characteristics

This study included 20 adults with HFA (10 males, 10 females) and 20 NC (10 males, 10 females). The mean age was 30.5 ± 7.8 years in HFA and 30.7 ± 7.8 years in NC, with an insignificant group difference at *p* = 0.920. The maximum age was 51 years in HFA and 48 years in NC, while the minimum age was 20 years in HFA and 22 years in NC. There was little group difference in total IQ score, *p* = 0.080, with 105.4 ± 11.2 points in HFA and 111.6 ± 10.4 points in total WAIS-IV score in the NC group with a maximum IQ of 125 points in HFA and 132 points in NC, while the minimum IQ was 88 in HFA and 99 in NC. The groups differed significantly on the autism-specific self-report psychometric tests AQ (37.9 ± 4.4 in HFA versus 13.7 ± 7.4 in NC) and EQ (18.0 ± 8.3 in HFA versus 46.5 ± 13.0 in NC), each at *p* < 0.001.

### Nanopore sequencing

#### Quality control

The mean coverage of the MAGEL2 sequence across all samples was 39.244 ± 30.140. The fragment chr15:23639316–23,647,088 showed a lower mean coverage of 38.832 ± 28.499 than the fragment chr15:23647429–23,651,466 with a mean coverage of 49.978 ± 43.489. Some variability in mean coverage between samples is evident from the distribution plot (Supplementary Material S2A). In the HFA group, there were two outliers with apparently higher coverage, which was also reflected in higher standard deviation in the HFA group (compare Table 1 with coverages in group comparison). However, there was no statistically significant group difference in mean coverage between HFA and NC in neither fragment. There was an almost constant coverage across individual CpG positions within each of the two fragments (see Supplementary Material S2B). Table 1 summarizes the basic coverage statistics.

**Table 1:** Coverage statistics for both fragments chr15:23639316–23647088 and chr15: 23647429–23651466 in group comparison

							t-test (coverage ∼ group)				
MAGEL2	**Genomic ranges (chr15:)**	**M**	**SD**	**Min**	**Max**	**t**		**df**		**p**	
Fragment 1	23639316–23647088	38.832	28.499	8.841	159.189	−0.320		30.479		0.751	
HFA		40.289	35.275	9.5373	159.189						
Control		37.375	20.454	8.841	78.119						
Fragment 2	23647429–23651466	49.978	43.489	7.5694	246.514	−0.252		28.362		0.803	
HFA		51.731	55.385	14.764	246.514						
Control		48.226	28.429	7.5694	100.306						

#### Sequence analysis

We found no obvious group-dependent clustering of snvs in either group. hfa and nc share most of the detected snvs, some of which were also classified as common within dbSNP [25]. Not a single variant was found to be relevant compared to ClinVar database. Focusing on the MAGEL2 regulatory units corresponding to ORegAnno, there were further no relevant group differences in the large regulatory relevant region OReg 0022988 (chr15:23642152–23643102), which is a binding site for the transcription factor CTCF. In the smaller regulatory regions OREG 1375344 (chr15:23645311–23645722, CTCF binding site), OREG 0157578 (23647924–23647934, SP1 binding site) and OREG 1375345 (23648972–23649252, CTCF binding site) there were only a few single SNPs in individuals of the HFA group but none in the NC group. None of these SNPs is known to be associated with autism spectrum disorders. Supplementary Material S3 provides a visualization of the SNPs detected across the entire gene sequence in group comparison, while Supplementary Material S4 lists the individual SNPs found within the gene regulatory relevant regions in tabular form.

#### Methylation analysis

We analyzed a total of 291 CpG positions across the captured sequence, 199 within fragment 1 spanning chr15:23639316–23647088 and 71 within fragment 2 (chr15:23647429–23651466). The remaining CpGs (*n* = 21) were in the range between the internal guides, which should not be evaluated for methodological reasons as it is insufficiently covered (see Supplementary Material 2B).

**Fig. 1:**
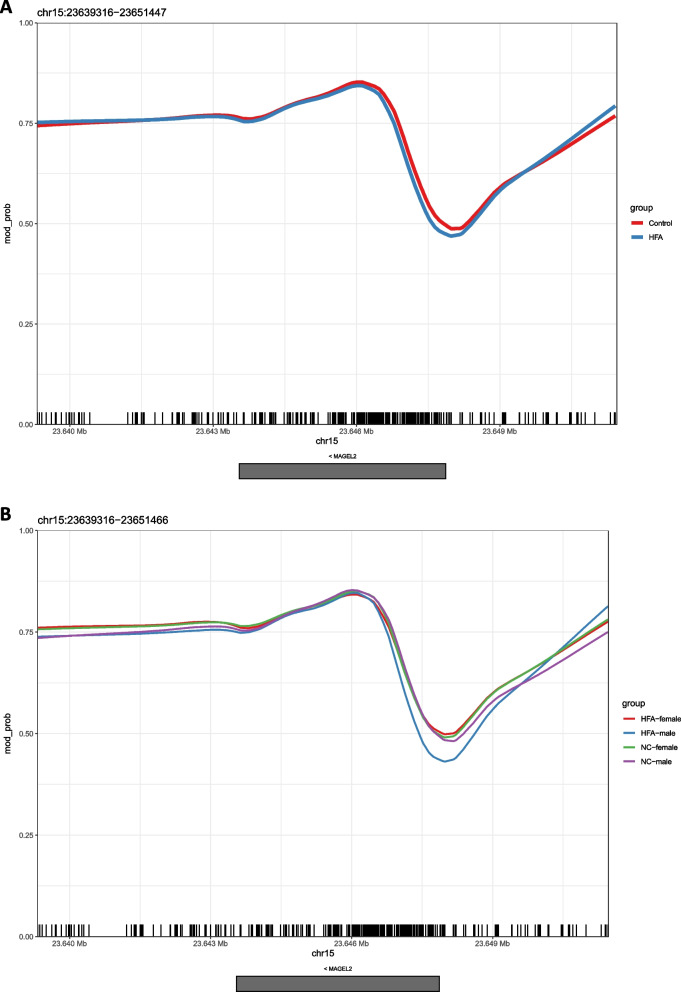
Figure 1**A** shows the 5mC modification probability across the entire MAGEL2 sequence including the regulatory construct. The MAGEL2 exon is represented by a gray box below the x-axis. While the thin lines in the figure show individual long reads, the thick lines represent the aggregated trend across all reads color-coded by group (blue - HFA, red - NC). While methylation was stable across the entire gene in the group comparison, a scissor-like drift of individual reads was observed in the region around the TSS of MAGEL2, where one part of the reads was hyper- and the other part clearly hypomethylated. Figure 1**B** shows the 5mC modification probability according to the scheme explained in Figure 1, but now in a differentiated comparison of the groups separated by sex (HFA-female - red, HFA-male - blue, NC-female - green, NC-male - purple). In the area around the MAGEL2 TSS, hypomethylation is clearly visible in the male HFA compared to the other groups

Figure 1A shows a so-called spaghetti plot of the entire MAGEL2 gene and its gene regulatory units. Each thin line within the plot represents the methylation pattern of an individual read across the genomic region, while the thick lines represent the aggregated trend, both color-coded by group. The height or depth of the peaks represents the degree of methylation, with consistent patterns across multiple reads indicating robust methylation. For most of the captured sequence, there were no apparent group differences in methylation. However, in the MAGEL2 promoter region within fragment 2 around the TSS (first base of exon 1, reverse strand, chr15:23647867; corresponding to the right end of the gray box in Fig. 1A representing the exon), there was a scissor-like drift in the methylation of individual reads, with one slightly hypermethylated and one distinctly hypomethylated fraction, but no clear tendency in group membership. Only at that region also the aggregated trends deviated slightly with otherwise extreme robust methylation.

Statistical analysis of differential methylation did not reveal any significant differentially methylated regions (DMRs). Two DMLs found (chr15:23647239 and chr15:23647248) were considered artifacts, as they were both located in the methodological blind region between the two inner crRNAs (chr15:23647088–23647429), which can also be seen as an interruption in the individual long reads in Fig. 1A.

Given the observed drift of single read methylation curves in the MAGEL2 promoter region, which did not represent a group-specific effect, we further examined the groups individually for sex differences (four groups, HFA-male, HFA-female, NC-male, NC-female, *n* = 10 subjects each). This showed an evident hypomethylation in a region around the MAGEL2 transcription start site (TSS) in male HFA subjects compared to female HFA subjects and controls of both sexes (Fig. 1B), whereas large proportions of the gene again showed constant methylation between the four groups. While differential methylation analysis between the NC group by sex (NC-male vs. NC-female) did not reveal any DMLs or DMRs (as expected from the graphical output), differential methylation analysis of the HFA group by sex (HFA-male vs. HFA-female) revealed a 300 bp differentially methylated region (chr15:23647640–23647939, mean difference = 5.3%, SE (diff) 1.1%, areaStat = −86.438) within the MAGEL2 promoter region including CpGs 72 bp up- to 227 bp downstream the TSS (chr15:23647867; 5’−3’ direction). While the male HFA group showed a mean methylation of 39.9%, the female HFA group showed a mean methylation of 45.2% across 18 CpGs within the 300 bp DMR. The results of the single CpG differential methylation loci analysis across the DMR and in relation to the TSS are shown in Table 2. Since it is difficult to estimate false discovery rates (FDR) for entire regions, the DSS tool does not provide FDR or p-values for DMRs, only for individual CpGs. Therefore, Table 2 presents the results from the CpGs within the DMRs identified by the callDML function in the first step. The areaStat parameter in DSS callDMR represents the sum of Wald test statistics (obtained by callDML) across all CpG sites within a DMR. Although it does not have a direct biological meaning, the areaStat combines both the “height” (magnitude of changes) and “width” (number of CpG sites) to rank DMRs. A larger areaStat suggests a region is more likely to be differentially methylated, which is useful for ranking potential DMRs. However, in our study, this metric is less relevant as only one DMR met the criteria outlined in the methods section. If no DMR was found by DSS callDMR function according to the criteria (see Methods), no statistics (e.g., means, areaStat) are output. As such, for those comparisons where no significant DMRs were identified, we cannot provide comparative test statistics.

**Fig. 2:**
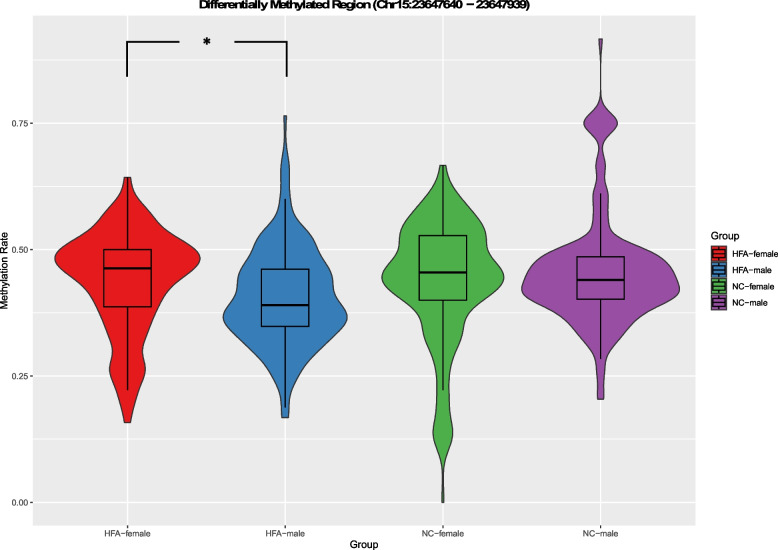
Figure 2 shows the overall distribution of 5mC modification probability data across all CpGs within the DMR (chr15:23647640–23647939) compared by group ∼ sex in a combined box and violin plot, with the violin plot providing additional information on distribution shape and density. Only in the HFA-female vs. HFA-male comparison, the region chr15:23647640–23647939 was found to be differentially methylated by the DSS callDMR function (indicated by an asterisk), while no DMRs were found in the other pairwise comparisons

Investigating the groupwise distributions of mean 5mC modification probabilities across all CpGs within the DMR chr15:23647640–23647939 by combined box- and violinplots (Fig. 2), one can see an overall similarity in scatter within the same sexes with males tending to hypomethylation compared to females. However, this effect was significantly more pronounced in the male HFA than the male NC group with a median below the Q1 level of the NC-males. In the NC-male group there were more extreme outliers towards hypermethylation, but however also more outliers towards hypomethylation than in the HFA-males. Compared to the HFA-female group the HFA-male median corresponded to the level of HFA-female Q1.

**Table 2:** Table 2 presents the sex-wise mean 5mC modification estimates at single CpG positions within the DMR (chr15:23647640–23647939) in the HFA group. These results were derived from differential methylation analysis at single loci using DSS callDML function, which the callDMR function is based on

CpG (chr15:)	Relation to TSS (chr15:23647867)	Mean Methylation HFA group		diff	SE (diff)	stat	pval
		male	female				
23647640	−227	0.404	0.459	0.055	0.012	4.494	< 0.001
23647644	−223	0.403	0.458	0.056	0.012	4.677	< 0.001
23647651	−216	0.404	0.458	0.055	0.012	4.642	< 0.001
23647662	−205	0.404	0.458	0.055	0.012	4.752	< 0.001
23647669	−198	0.404	0.458	0.055	0.012	4.752	< 0.001
23647683	−184	0.401	0.454	0.054	0.011	4.682	< 0.001
23647689	−178	0.398	0.450	0.053	0.011	4.749	< 0.001
23647698	−169	0.395	0.447	0.053	0.011	4.800	< 0.001
23647701	−166	0.394	0.447	0.052	0.011	4.842	< 0.001
23647710	−157	0.394	0.447	0.052	0.011	4.842	< 0.001
23647737	−130	0.399	0.450	0.051	0.010	5.209	< 0.001
23647776	−91	0.400	0.451	0.051	0.010	5.280	< 0.001
23647821	−46	0.400	0.451	0.051	0.010	5.014	< 0.001
23647878	+ 11	0.399	0.451	0.052	0.011	4.791	< 0.001
23647883	+ 16	0.399	0.451	0.052	0.011	4.705	< 0.001
23647893	+ 26	0.398	0.451	0.052	0.011	4.617	< 0.001
23647928	+ 61	0.397	0.450	0.053	0.010	5.126	< 0.001
23647939	+ 72	0.396	0.448	0.051	0.012	4.464	< 0.001

Given the uneven distribution of coverages between the individual samples mentioned above, we were prompted to consider whether this might be a coverage-related artefact. Accordingly, we repeated the analysis steps by excluding individual samples falling below different minimum mean coverages. At reduced group sizes with a minimum coverage of 10x (*N* = 39, NC female group *n* = 9) as well as at an underlying minimum coverage of 15x (*N* = 36, HFA male *n* = 8, NC male *n* = 9, NC female *n* = 9), there were no apparent effects on the methylation probabilities. A minimum coverage of 20x (*N* = 28, HFA male *n* = 5, HFA female *n* = 9, NC male *n* = 8, NC female *n* = 6) revealed isolated visible differences in the methylation probability. However, there was still an evident difference between the HFA-male group and the others in the DMA described above. Figure S5 in the Supplementary Material compares the methylation plots as shown in Fig. 1, considering different minimum coverages.

## Discussion

In this pilot study, we examined the entire MAGEL2 gene in adults with HFA using a long-read nanopore targeted sequencing (nCATS) approach and compared the results for sequence variation and methylation (5mC) with sex- and age-matched NC.

MAGEL2 sequence analysis did not reveal any significant differences between the groups. In particular, we did not identify any variants associated with syndromic autism spectrum disorder, for example, within nucleotides c.1990–1996, which represents a sequence of seven cytosines acting as a mutation hotspot in SYS [26], nor any novel variants that would have clustered in a group-dependent manner.

While methylation (5mC modification) was stable and consistent between groups over the vast majority of the MAGEL2 gene sequence, there was an apparent drift-apart of individual read methylation trends around the TSS within the MAGEL2 gene promoter region. When the two groups were further separated by sex, a differentially methylated region of 300 bp emerged at this site when comparing HFA males to HFA females and both sex controls. There was a statistically significant decrease in 5mC modification around the TSS in HFA males.

Of course, these data on differential methylation of the MAGEL2 promoter region between the sexes must be interpreted with great caution due to the small number of cases in this pilot study (*n* = 10 per group when analyzed separately by sex). Looking at the scatter of the data (Fig. 2), there seems to be a global difference between the sexes with a tendency towards higher average methylation within the DMR in females and lower in males, an effect that seems to be more pronounced in the male HFA group. However, we find this effect particularly impressive given the otherwise extremely stable methylation between the groups across the entire MAGEL2 gene and a total of 291 CpG positions that we analyzed and visualized using the long-read sequencing approach.

Sex differences in the phenotype of ASD are known. For example, females with autism show a greater ability than males to form, understand, and maintain relationships and, in particular, to adapt their behavior to different social situations [7]. However, this means that high-functioning females with autism often remain undiagnosed longer than males, which has implications for comorbidity profiles and treatment success. Sex differences in MAGEL2 function are also known, at least from animal models. In addition to the sex-specific endocrine abnormalities observed in Magel2-deficient mice [27], recent animal studies of MAGEL2 have also found altered spatial learning and social recognition memory in male Magel2 KO mice, but not in female Magel2 KO mice [5, 28]. This may be due in part to sex-specific expression of oxytocin receptors, as has been found in mice [29].

However, to our knowledge, this study is the first to systematically examine the 5mC modification across the entire MAGEL2 gene and the first to demonstrate sex-specific epigenetic MAGEL2 differences in humans with ASD. Could it be that the subtleties of the known sex differences in autism are also influenced by epigenetic regulatory differences in MAGEL2? In particular, the influence of MAGEL2 in hypothalamic signaling pathways in PWS has been studied, but the role of MAGEL2 at the cellular level is not fully understood. According to available publications, MAGEL2 function is associated with cell surface processes. It has been shown to be involved in the regulation of secretory granules (SG), which are characteristic of neuroendocrine cells and serve as organelles for the processing and secretion of hormones and neuropeptides. Enzymes required for processing include proprotein convertases such as PCSK1 and 2, which were found to be severely downregulated in hypothalamic tissue of MAGEL2 knockout mice, ultimately reducing the amount of mature SGs in neurons and circulating bioactive hormones [30]. An altered function of the oxytocin system is often discussed as a possible consequence.

The relationship between MAGEL2 and the oxytocin system has generally been the focus of previous reports on MAGEL2. Oxytocin is produced in the hypothalamus, the region where Magel2 is most abundant [31]. Disruption of MAGEL2 has been shown to significantly reduce oxytocin levels in the mouse hypothalamus [30]. Finally, oxytocin stimulates the expression of numerous genes involved in neurite outgrowth and synapse formation, which have been shown to be impaired in MAGEL2 deficiency [32]. This is thought to be one reason why Magel2-deficient mouse models have shown the aforementioned changes in behavioral phenotypes. Some behavioral abnormalities could be compensated by oxytocin administration [28, 33]. For example, oxytocin administration has been shown to partially reverse hippocampal alterations and social memory deficits found in animal models [5]. Recently, Magel2-KO female mice, which were previously shown to lack the social deficits observed in Magel2-KO males, were characterized by a different trend in the expression of the oxytocin receptor gene (OXTR) compared to male mice [34].

According to the ORegAnno database, the DMR (chr15:23647640–23647939) we found here in the sex comparison includes the gene regulatory units OReg 01537577 (chr15:23647901–23647911) and OReg 01537578 (chr15:23647924–23647934) and partially also OReg 0157579 (chr15:23647935–23647945). All are binding sites for the transcription factor SP1 (Specificity Protein 1). SP1 is an ubiquitously expressed transcription factor, that regulates the expression of a large number of genes and is associated with a variety of cellular processes [35]. Modification of 5mC, particularly within the gene promoter region, influences gene regulation. Hypomethylation within these regions is generally thought to promote transcription factor binding and transcriptional up-regulation, but this is not necessarily the case for all genes. In the case of the MAGEL2 gene, to our knowledge, the influence of 5mC methylation and SP1 on the regulation of gene expression has not been investigated in humans. However, in silico analysis suggested a dual activator or repressor role of SP1 in response to physiological and pathological stimuli in MAGE family genes [36]. Therefore, a counterregulatory mechanism is likely, i.e., that existing deficits in the oxytocinergic system are counteracted by altered epigenetic regulation of MAGEL2 and associated improved functionality of the neurotransmitter system.

In the case of OXTR, a sex influence on methylation is known from studies in humans with ASD [37]. However, the results of our nanopore pilot study of the OXTR gene showed no differences in OXTR sequence and methylation between individuals with HFA and controls, nor between the sexes [38]. Therefore, it may be concluded that the sex differences observed here for MAGEL2 in HFA have at least no influence on OXTR.

It has also been hypothesized that MAGEL2 interactions may play a role in RNA metabolism and regulation of mRNAs [39]. In a recently published study, MAGEL2 was investigated using proximity-based biotin identification (BioID). The proteins identified as proximal to MAGEL2 were found to be primarily involved in mRNA metabolism. MAGEL2 is thought to be particularly involved in the regulation of mRNAs modified by m6A methylation. Methylation of N6-methyladenosine (m6A) is the most common form of cotranscriptional modification in eukaryotic RNAs. Unfortunately, no RNA material was available for this study. However, it would certainly be interesting to investigate this in the future.

### Limitations

A major limitation of this study is the small sample size due to the pilot nature of the study. Underpowering cannot be ruled out, especially in differentiated subgroup analyses. Comparable previous studies for sample size estimation were not available. Data on nanopore long read sequencing, especially nCATS, in clinical samples are very scarce. Also, the MAGEL2 gene itself has not been studied in human case-control collectives, especially with respect to methylation, and not in non-syndromic ASD conditions, which limits the comparability of our pilot results with those of previous studies. Clearly, these initial results obtained with nCATS need to be replicated in independent cohorts and with larger sample sizes.

However, the differential methylation analysis algorithm chosen here has been described by the authors as particularly suitable for small sample sizes [40]. Previous comparative analyses showed that DSS with its count-based beta-binomial statistical model had one of the lowest observed type I error rates in 15 versus 15 samples (as comparable to this study size) and was shown to discriminate well when testing for 5% methylation differences as applied here (AUC of 0.867 in ROC analysis). DSS has been shown to outperform other approaches when the sample size is small, which could be demonstrated when the sample size was reduced to 5 versus 5 samples, with DSS still showing acceptable to good discrimination with an AUC of 0.797 [41].

Overall, it should be kept in mind that this is a pilot study that also aims to demonstrate the capabilities but also the limitations of third-generation sequencing approaches such as nCATS, and to identify trends for autism research from their application in clinical collectives.

As a novel technology, our target-based nanopore approach has methodological pitfalls that may have influenced these early results. Due to initial difficulties in achieving sufficient coverage of the entire gene, we decided to generate two fragments of the MAGEL2 gene with four crRNAs to optimize the result. Internal guides within the exon of MAGEL2 proved to be the best performing crRNAs during the establishment phase, which unfortunately was accompanied by a small blind spot within this relevant region. From independent comparative studies of nCATS with previous methods conducted in our laboratory, we have no evidence that the positioning of the guides affects the methylation results of adjacent gene regions [15]. Looking at the methylation data presented here, only very few CpG positions adjacent to the guides are affected in their coverage (compare Supplementary Materia S2B). The DMR detected between the HFA sexes was not affected by this. With generally different efficiency of nCATS [42] and hardly any experience in clinical collectives, the coverage of CpGs within the two MAGEL2 fragments was stable (see Supplementary Material S2B) and amounted to > 30 on average for both. Indeed, there was variability in coverage between samples, as well as comparatively low coverage remaining in a few samples (see Supplementary Material S2A), which may have affected the sensitivity of the 5mC modification estimates. The statistical model used for DMA has been described to account for coverage [24]. There was no statistically significant difference in coverage between groups. Furthermore, the exclusion of samples with low coverage (see Supplementary Material S5) also showed no apparent effect on group-dependent methylation. Overall, we consider methodological bias to be unlikely given the very constant methylation patterns over large parts of the gene, irrespective of the underlying minimum coverage.

Another important limitation of this study is the use of peripheral blood material for methylation analysis, which limits its relevance to central processes, whereas MAGEL2 shows predominantly cerebral expression. While the average overall methylation correlation between brain tissue and peripheral blood material proved to be reliable in a preliminary comparative study (0.86), a significantly poorer correlation was found for individual CpG sites between blood and brain tissue (20.8% at *p* < 0.05) [43].

In addition, our approach does not provide information on actual gene expression, so it is not possible to say to what extent the HFA sex differences found in methylation of the MAGEL2 promoter region actually influence MAGEL2 gene expression. This should be demonstrated in future studies, e.g. by RNA sequencing analyses.

Finally, it is important to remember that we studied individuals with HFA diagnosed in adulthood, which is only a subgroup of the overall autism spectrum. However, we know from other autism susceptibility genes that the methylation patterns of syndromic and non-syndromic autism conditions correlate [10]. Given the known difficulties in diagnosing HFA in adulthood, accurate diagnosis at recruitment is required. Guidelines (neither the outdated German S2 guideline nor the UK NICE guideline) provide clear recommendations on diagnostic tools for diagnosing HFA in adulthood. The Adult Asperger Assessment used here refers to a prospectively outdated diagnosis, but is comparable to the ICD-11 diagnosis of autism spectrum disorder without cognitive delay and language impairment.

## Conclusion

In conclusion, this pilot study of the MAGEL2 gene used the long-read nanopore sequencing method nCATS in adults with high-functioning autism within a case-control cohort. The results showed differences in MAGEL2 gene promoter methylation between males and females with HFA and NC, suggesting the potential for sex-specific epigenetic differences. It is important to note that this pilot study was conducted with a limited number of samples and therefore the results should be validated in larger, independent cohorts in the future, also using RNA sequencing data to further elucidate the functional significance of the observed methylation variance. Nevertheless, these findings highlight the need to consider sex differences in autism research, which may also pave the way for further research into biomarkers or pharmacological targets in this area.

## Supplementary Information

Supplementary Material 1.

## Data Availability

The data sets generated and analyzed in this study are available from the European Genome Phenome Archive (EGA) at https://ega-archive.org/studies/EGAS50000000508. The analysis code used here is publicly available at https://github.com/wietingj/nCATS_analysis.

## Abbreviations

AAA: Adult Asperger Assessment
AQ: Autism Quotient
ASD: Autism Spectrum Disorder
crRNA: CRISPR RNA
DML: Differentially methylated locus
DMR: Differentially methylated region
EQ: Empathy Quotient
HFA: High functioning autism
KO: Knock-out
MAGEL2: MAGE family member L2
nCATS: Nanopore Cas9 targeted sequencing
NC: Neurotypical control
OXTR: Oxytocin receptor gene
PAM: Protospacer adjacent motif
PWS: Prader-Willi syndrome
SG: Secretory Granules
SNV: Single nucleotide variant
SYS: Schaaf-Yang syndrome
tracrRNA: Trans-activating CRISPR RNA
TSS: transcription starting site
WAIS-IV: Wechsler Adult Intelligence Scale IV
