# Supplementary material for: Sex differences in MAGEL2 gene promoter methylation in high functioning autism - trends from a pilot study using nanopore Cas9 targeted long read sequencing
Source: BMC Med Genomics. 2024 Nov 29;17:279. doi: 10.1186/s12920-024-02053-9 (PMC11606058; doi:10.1186/s12920-024-02053-9)
Supplement: Supplementary file 1 — Supplementary Material 1. [file 12920_2024_2053_MOESM1_ESM.docx]

**SUPPLEMENTARY MATERIAL**

SUPPLEMENTARY MATERIAL S1

| ID | Sequence |  | Genomic position |
| --- | --- | --- | --- |
| 66_MAGEL2 | TTAGCGTCACTAATAAGAGT | AGG | Chr15:**23639316**-23639336 |
| 73_MAGEL2_Innen | GGAGTCGGAGGCTTACCCAT | CGG | Chr15:23647429-*23647448* |
| 74_MAGEL2_Innen | TCCACCTCCGGGGACACCGA | TGG | Chr15:23647107-*23647088* |
| 75_MAGEL_Ersatz65 | AGTGACCACTCAATGCGAGC | TGG | Chr15:**23651466**-23651447 |

**S1** Sequence, PAM motifs and Genomic positions of crRNA “guides” used for Cas9-mediated PCR-free enrichment

SUPPLEMENTARY MATERIAL S2

**S2** Coverage Statistics **- A** shows the individual mean coverage per sample color-coded by group, with two outliers in the HFA group. **B** shows the coverage at individual CpG positions according to their genomic position across all samples. Coverage within the two fragments (fragment 1 blue, 2 red) was stable, with fragment 2 showing a significantly higher mean coverage.

SUPPLEMENTARY MATERIAL S3

**S3** Visualization of the SNPs detected across the entire gene sequence in group comparison (SNPs HFA group above SNPs NC group). In addition, known gene regulatory units according to ORegAnno as well as common variants (common dbSNP) and clinically relevant variants (ClinVar) are shown.

SUPPLEMENTARY MATERIALS S4

| **OREG** | **Position** | **Function** | **SNP** | | **n per Group** | |
| --- | --- | --- | --- | --- | --- | --- |
|  |  | TF binding site: | GRCh38(chr15): | | HFA | NC |
| **0022988** | 23642152-23643102 | CTCF | g.23642262G>A | | 2 | 2 |
|  |  |  | g.23642294G>A | | 0 | 1 |
|  |  |  | g.23642564G>A | | 1 | 1 |
|  |  |  | g.23642573G>A | | 1 | 0 |
|  |  |  | g.23642592C>T | | 0 | 1 |
|  |  |  | g.23642630G>A | | 1 | 0 |
|  |  |  | g.23642661G>A | | 1 | 0 |
|  |  |  | g.23642699T>C | | 1 | 0 |
|  |  |  | g.23642881G>A | | 0 | 1 |
|  |  |  | g.23642907C>T | | 0 | 1 |
| **1375344** | 23645311-23645722 | CTCF | g.23645647G>A  g.23645664C>T | | 1 | 0 |
|  |  |  |  |  | 1 | 0 |
| **0157577** | 23647901-23647911 | SP1 | - | | - | - |
| **0157578** | 23647924-23647934 | SP1 | g.23647928C>T | | 1 | 0 |
| **0157579** | 23647935-23647945 | SP1 | - | | - | - |
| **1375345** | 23648972-23649252 | CTCF | g.23649085G>A | | 1 | 0 |
| **TOTAL** |  |  |  |  | 11 | 7 |

**S4** List of the individual SNPs found within the gene regulatory relevant regions according to ORegAnno by group.

SUPPLEMENTARY MATERIAL S5

**S5** compares the methylation plots as shown in Figure 2, taking into account different minimum coverages.
